# Finite element analysis and a pilot study of different fixation constructs for Danis-Weber A and B lateral malleolus fractures

**DOI:** 10.1186/s12891-023-07115-0

**Published:** 2023-12-19

**Authors:** Shuming Huang, Junkun Zhu, Hailin Xing, Ruifeng Yang, Jifei Ye, Fang Ye, Quanzhou Wu, Shuhua Lan

**Affiliations:** 1 Department of Orthopedic Surgery, Lishui Hospital, Zhejiang University School of Medicine, The Fifth Affiliated Hospital of Wenzhou Medical University, Lishui Municipal Central Hospital, Lishui, Zhejiang 323000 People’s Republic of China; 2 Department of Orthopedic Rehabilitation, Lishui Hospital, Zhejiang University School of Medicine, The Fifth Affiliated Hospital of Wenzhou Medical University, Lishui Municipal Central Hospital, Zhejiang323000, Lishui, People’s Republic of China

**Keywords:** Finite element, Ankle fracture, Fibular fracture, Osteosynthesis, Fracture fixation

## Abstract

**Background:**

Displaced lateral malleolus fractures are typically stabilised through open reduction and internal fixation. The biomechanically and clinically efficacy of locking plates and lag screws, particularly in Weber A and B distal fibular fractures remains a subject of contention. This study examines two locking plate designs for lateral malleolus fractures, evaluating their performance with and without interfragmentary screws using finite element models.

**Methods:**

Utilising CT images of a healthy adult male volunteer, a three-dimensional finite element model was constructed. The Fibula-specific Flank Multiaxial Locking Anatomic Plate (FMLP) and the Conventional Locking Plate (CLP) were subjected to stabilisation, both with and without an interfragmentary screw, mimicking the Danis-Weber A and B lateral malleolus oblique fracture fixation. Loads of 140 N and 70 N, equivalent to 20% of the body weight, were applied to simulate the single-leg and two-leg standing conditions in the axial direction. The von Mises stress (VMS) distributions and element displacements were subsequently analyzed.

**Results:**

In the Danis-Weber A fracture model group, the FMLP with an interfragmentary screw fixation exhibited the lowest peak VMS values: 51.9 MPa in the fibula, 89.0 MPa in the plate, and 61.3 MPa in the screws for simulating single-leg conditions. Under two-leg standing conditions, these peak VMS values decreased to 25.9 MPa in the fibula, 44.5 MPa in the plate, and 30.6 MPa in the screws, respectively. Furthermore, the overall structural peak displacements during single-leg standing for both Weber-A and B fractures with different implants ranged from 1.61 to 2.54 mm. While standing on two feet, the ranged was from 0.80 to 1.27 mm. An interfragmentary screw at the oblique fracture site resulted in reduced the peak value of VMS in the fibula, plate, screws, consequently decreased the overall structural displacement for FMLP and CLP fixation in lateral malleolus fractures.

**Conclusions:**

The current finite element analysis (FEA) demonstrates that FMLP exhibits superior mechanical characteristics in Danis-Weber A and B lateral malleolus fractures compared to CLP. The inclusion of an interfragmentary screw, combined with locking plate design, enhances stability for simple oblique distal fibular fractures. The FMLP presents itself as potential as an alternative for lateral malleolus fractures from a biomechanical perspective. Nevertheless, further verification of these results is imperative through subsequent clinical studies.

## Background

Lateral malleolus fractures, also recognised as distal fibular fractures, exhibit an incidence rate of 74 per 100,000 individuals annually, displaying a bimodal age distribution with peaks observed in younger males and postmenopausal females [1]. Typically, these fractures are categorised using either the Danis-Weber or the Arbeitsgemeinschaft für Osteosynthesefragen Foundation/Orthopaedic Trauma Association (AO/OTA) classification systems, both of which consider the fracture's location in relation to the tibiofibular syndesmosis. The predominant types of distal fibular fractures are type A infra-syndesmotic and trans-syndesmotic type B, constituting up to 90% of all cases [2, 3]. Fractures that are intra-articular, displaying > 2 mm displacement, and exhibiting instability through rotation, shortening, or oblique necessitate open reduction and internal fixation (ORIF) to mitigate the risk of posttraumatic complications [4, 5].

Intramedullary fixation stands as an alternative in cases where poor soft tissue quality is compromised, potentially mitigating complications associated with incisions and irritation arising from metalwork prominence. However, certain compromises may be necessary to diminish the risk of anatomical fracture [6–8]. Traditional fixation methods, such as K-wires and tension band wiring, offer a degree of fixation for distal fibular fractures, but they often lack sufficient mechanical support and anti-rotation stability [9]. Plate osteosynthesis emerges as most commonly employed modality for the surgical management of lateral malleolus fractures [1, 6]. In recent decades, fixation techniques have progressed, encompassing lag screw fixation for oblique fractures, posterior and posterolateral antiglide (or buttress) plating fixation, and the advent of low-profile, anatomically pre-contoured, and locking of distal fibular plates [1, 4, 10–12]. These techniques have enhanced the stability of internal fixation and reduced soft tissue irritation. However, consensus regarding the optimal fixation method and the ideal implant remains elusive. Traditionally, the one-third tubular buttress plate has been utilised to minimise the risk of wound issues and screw penetration into the talofibular articular. Nonetheless, this plate may introduce hardware-related peroneal tendon complications [9, 12]. Furthermore, one-third of the tubular plate may prove inadequate for reliable fixation reliable fixation, particularly in the distal fracture segment. Operative fixation of complex distal fibular fractures, especially those involving osteoporosis, comminution, or more distal fractures, poses challenges challenging due to limited screw purchase and unicortical fixation [13]. Consequently, anatomically pre-contoured lateral locking plates are gaining popularity in the treatment of lateral malleolus fractures [1, 10, 13, 14]. Conventional lateral anatomic plates (CLP), typically designed for low-profile and minimal hardware prominence with a single row of locking screw holes, face limitations in the number of screws available for fixation in distal fractures. Attempts to increase the number of screws in the distal extent through a double row of locking holes in CLP may lead to soft tissue irritation and an increased burden on local skin covering on the lateral malleolus.

**Fig. 1:**
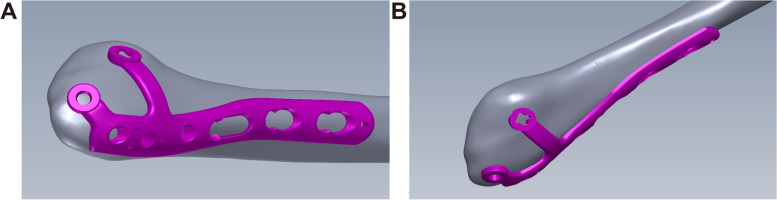
A schematic illustrating the Fibular Medial Locking Plate (FMLP) applied to the distal fibula. **A** Lateral view; **B** Front view

The current investigation involves the design and development of a novel Flank Multiaxial Locking Anatomic Plate (FMLP) intended for posterolateral fixation and multidirectional locking. This innovative plate incorporates distal and an anterolateral wings (Fig. 1). Notably, this approach has received approval for a national invention patent (number 201410513599.9) and a national medical device registration change application (number 20153460877). The FMLP's primary body can be situated along the posterior lateral ridge of the fibula, thereby reducing the risk of interference with the peroneal tendon and facilitating the insertion of long screws from the posterolateral to the anteromedial direction. The dual wings of the FMLP enhance the number of distal fixation screws while maintaining a low profile and minimising hardware prominence. Additionally, the axes of all the locking holes are non-parallel, with the combination of the main plate and double wing branches designed to be soft tissue-friendly, ensuring effective fracture fixation in multiple planes.

Conventional biomechanical experiments often yield mechanical parameters with inherent limitations. Notably, the accuracy of measurements can be influenced by variations in bone quality among diverse cadaveric specimens. Finite Element Analysis (FEA) in the context of the ankle joint is akin to physical testing in terms of effectiveness and consistency. Clinical practice routinely involves the fixation of oblique distal fibula fractures with interfragmentary lag screws and plates. However, the potential advantages of combining a locking plate with an interfragmentary lag screw remain controversial in both biomechanical and clinical studies [15, 16]. The current study aims to test the hypothesis that FMLP surpasses Conventional Locking Plate (CLP) in stabilising unstable fractures. A computer-simulated FEA was executed to investigate the biomechanical properties of two different locking anatomic plates, with or without interfragmentary screw constructs, for Danis-Weber A and B type fractures. The results furnish biomechanical evidence to inform the selection of appropriate implants.

## Methods

### Establishment of the finite element analysis model of the lateral malleolus

A single healthy male volunteer, aged 32, with a normal left ankle, standing at a height of 172 cm and weighing 70 kg, participated in this study. The research protocol received approval from the Ethics Committee of Lishui Municipal Central Hospital, and the volunteer provided informed consent before study participation. The individual had no history of ankle-related ailments and underwent radiographic examination of the left lower extremity to confirm the absence of injury or deformity. The left ankle of the volunteer was imaged in the neutral unloaded position using a 256-row spiral computed tomography (CT) scanner, operating at a voltage of 120 kV, a slice thickness of 0.9 mm, and an interval of 0.45 mm. The resulting cross-sectional CT images were stored in Digital Imaging and Communications in Medicine (DICOM) format and imported into Mimics 16.0 (Materialise, Leuven, Belgium) for the initial three-dimensional (3D) geometry model reconstruction of the distal fibula. Subsequently, the Stereolithography (STL) format file of the distal fibula was integrated into Geomagic Studio 2014 (Raindrop Geomagio Inc, Morrisville, N.C, USA) for further processing, involving denoising, smoothing, and exact surface fitting to generate an accurate geometric solid model. The final model was exported as an STP file.

**Fig. 2:**
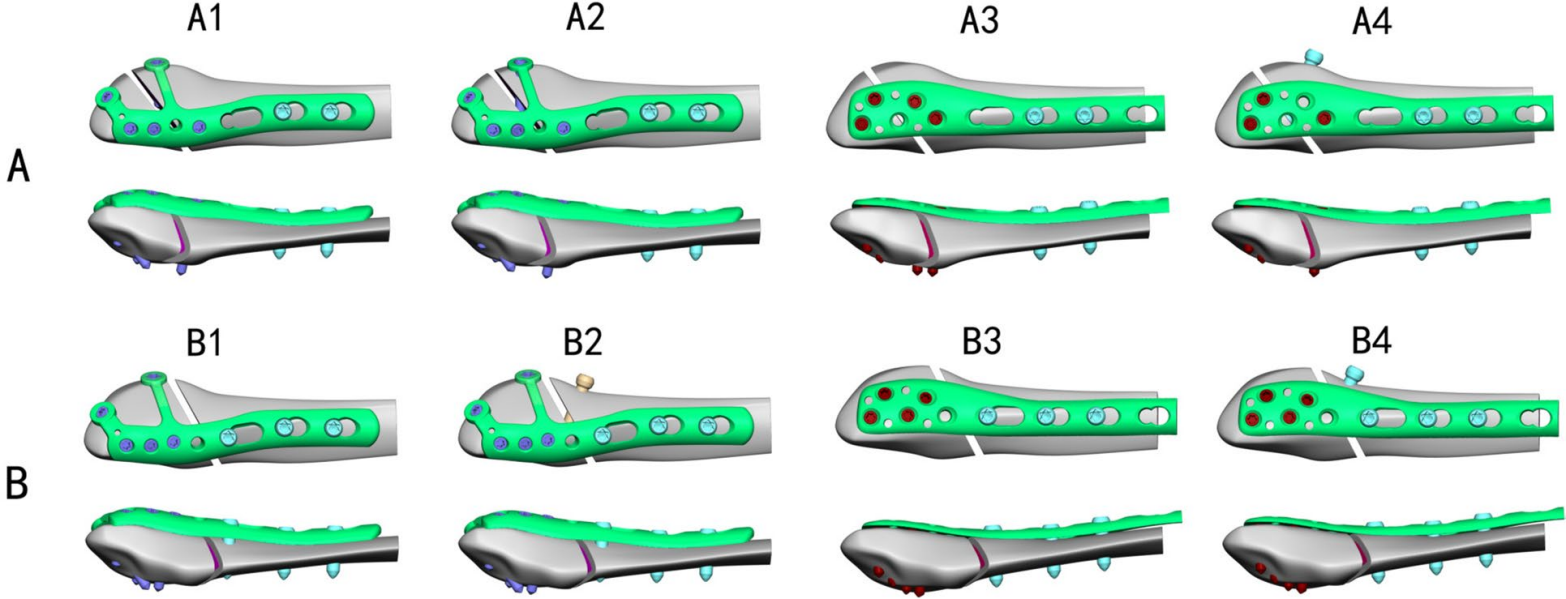
Various geometric models for internal fixation were developed for lateral malleolus fractures of Danis-Weber A and B types. A1: FMLP for the type A fracture model. A2: FMLP with an interfragmentary screw for the type A fracture model. A3: Conventional Locking Plate (CLP) for the A fracture model. A4: CLP with an interfragmentary screw for the type A fracture model. B1: FMLP for Danis-Weber type B fracture. B2: FMLP with an interfragmentary screw for the type B fracture. B3: CLP for the type B fracture. B4: CLP with an interfragmentary screw for the type B fracture

Various types of internal fixation constructs/implants, including Flank Multiaxial Locking Anatomic Plate (FMLP) and Conventional Locking Plate (CLP) with or without an interfragmentary screw, were established to simulate fixation for Danis-Weber A and B lateral malleolus oblique fractures. The oblique fractures were intentionally from anteroinferior to posterosuperior at an angle of a 45° to the fibula surface [17]. In the models with interfragmentary screws, a screw was employed to stabilize the fracture gap at a 90° angle to the fracture line, after which the plate was inserted and secured. For Danis-Weber type A fractures, four fixation models were established: (1) A1 group, FMLP without an interfragmentary screw. (2) A2 group, FMLP with an interfragmentary screw. (3) A3 group, CLP without an interfragmentary screw. (4) A4 group, CLP with an interfragmentary screw. Similarly, four fixation models were established for Danis-Weber type B fractures: (1) B1 group, FMLP without an interfragmentary screw. (2) B2 group, FMLP with an interfragmentary screw. (3)B3 group, CLP without an interfragmentary screw. (4) B4 group, CLP with an interfragmentary screw (Fig. 2).

Subsequently, the Stereolithography (STL) format file of the internal fixation geometry model for lateral malleolus fracture underwent importation into the pre-processing software Hypermesh13.0 (Altair, CA, USA) for mesh division. The finite element meshing, secondary processing, and analysis were executed in MSC.Patran/Nastran 2012 (MSC, USA). Employing 4-node tetrahedral 3D elements, chosen for their suitability in nonlinear geometric analysis, the models were subjected to mesh convergence tests to ensure a sufficiently refined element discretization. Mesh convergence tests confirmed that the deviation was less than 5% adhering to the convergence criterion, aligning with previous studies demonstrating minimal impact on results with mesh refinement up to a certain extent. The mesh quality in this model is commendable, with a relatively uniform tetrahedral side length and deformity-free meshes, meeting the prescribed standards for mesh quality and convergence. Details regarding the number of nodes and elements for each group are presented in Table 1.

**Table 1:** Number of nodes and elements for each implant and fracture model group of the distal fibula

Model	Node	Element
A1	76756	393965
A2	79433	408816
A3	70098	330880
A4	69497	327679
B1	76727	394579
B2	77419	397873
B3	90058	452090
B4	85170	420484

### Material parameters

In this study, the material mechanical properties of the materials were derived from previous literature [18–24]. Ti-6Al-4 V was employed to simulate the implants, including plates and screws. The fibula, plate, and screws were assumed to be isotropic, homogeneous, and to exhibit continuous linear elasticity. Locking screw heads of the fixation were embedded into the plate and the screws were completely tied and bonded to the plate. To prevent screw loosening, an axial force of 2.5N was applied as preload to the screws [22]. Frictional contact conditions were established between the fibula bone and the implants in all models. These frictional contact interactions adhered to a well-established and validated contact setup method, as describedin previous studies [18, 22, 24]. Interfaces between the screw-bone and plate-bone interaction were modelled as contacts with coefficient of frictionset at 0.3 [18, 24]. Fxation screws were inserted into the fibula bone tissue in various fracture and fixation models using frictional contact and coupling conditions. All nodes located on the surface of the screw and the fibula bone screw hole were considered to be fully coupled conditions. The parameters for each structural material used in the analysis are detailed in Table 2.

**Table 2:** Properties of the various components in the finite element models

Component name	Young’s modulus (MPa)	Poisson’s ratio	References
Fibula cortical bone	7300	0.30	[21]
Fibula cancellous bone	1100	0.26	[20]
Plate	110000	0.30	[19, 20, 23]
Screw (Ti-6Al-4 V)	113800	0.34	[18, 24]

### Loading and boundary conditions

**Fig. 3:**
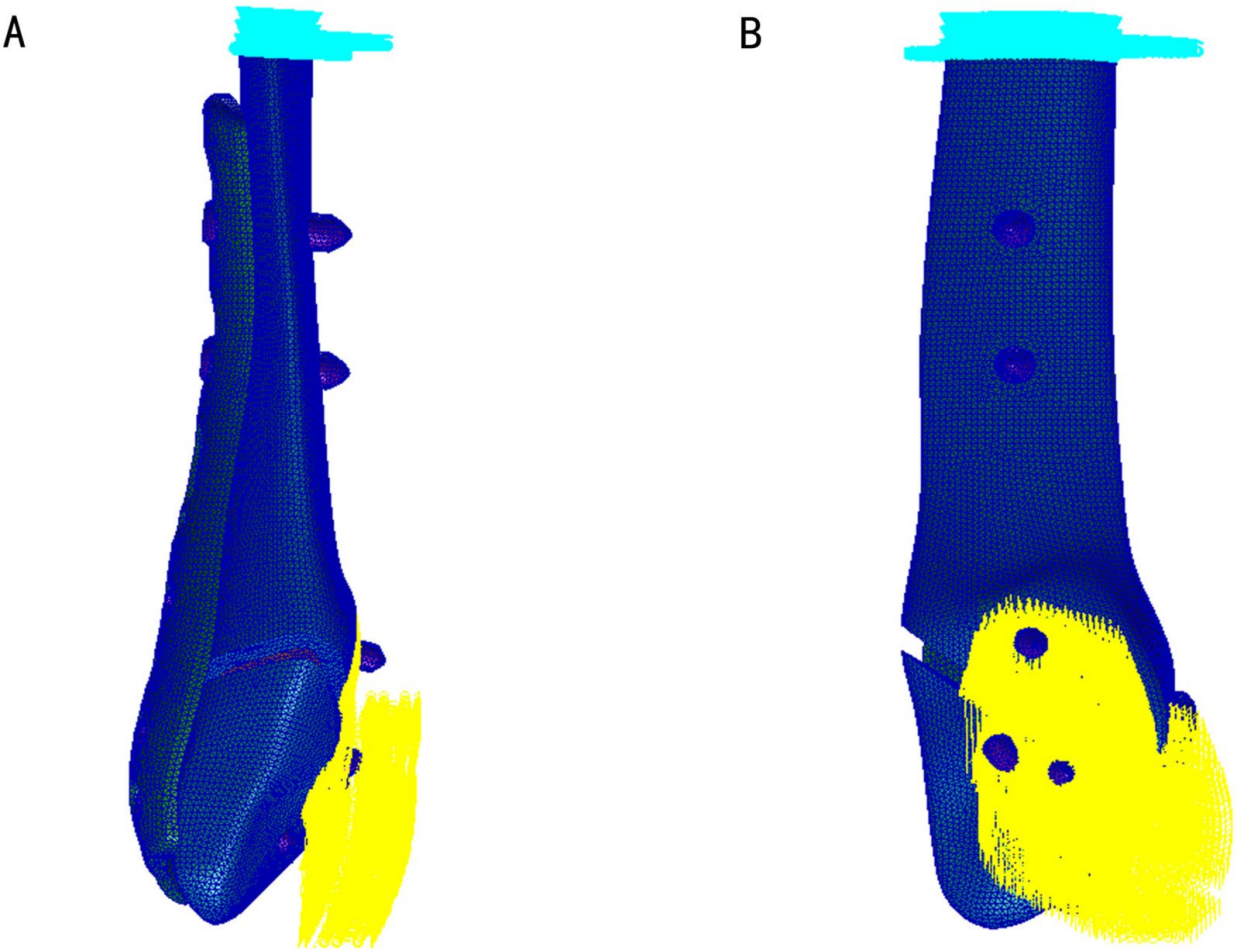
Boundary conditions of fibula fixation (A1 as an example)

Boundary constraints were applied to the internal fixation structure models of the fibula [25–27]. Whether standing on one foot or two, the weight of the human body is transmitted to the ankle and borne by both the tibia and fibula simultaneously. In this study, only static axial loading was considered, as it represents the predominant loading scenario for the fibula in weight-bearing conditions. Previous studies indicate that the tibia bears approximately 80% of the body weight, while the fibula supports around 15–20% of a person’s weight during single-leg standing [28–30]. All the models were assessed under physiological conditions encompassing both single-leg and two-leg stances. Hence, volunteer with a body weight of 70 kg (700 N) was employed, with loads of 140 N and 70 N respectively, equivalent to 20% of the body weight, to simulate the axial direction in single-leg and two-leg standing conditions. Following the principle of action and reaction force, all nodes of the proximal section of the fibula were constrained in all directions, and loads were applied to the joint contact surfaces corresponding to the tibiofibular joint at the distal end of the fibula to simulate the stress state of the fibula. The negligible effect of gravity and the simplification of models by neglecting muscle forces were considered [19, 31–34]. These conditions, while simplifying the models, do not compromise the validity of the results, as the objective is to compare different models under consistent conditions. Consequently, the presented results can be deemed credible and objective. The boundary condition constraint (illustrated with Weber-A1 as an example) is depicted in Fig. 3.

### Stress analysis and measurement

The von Mises stress, a commonly employed scalar quantity in the realms of mechanical engineering and material science, served as the criterion for assessing the performance of the plates [35, 36]. This stress criterion enables the evaluation of the yield or failure of the plates under complex loading conditions.

### Data analysis

The values derived from finite element analysis are precise and unique.

## Results

### von Mises stress distribution

**Fig. 4:**
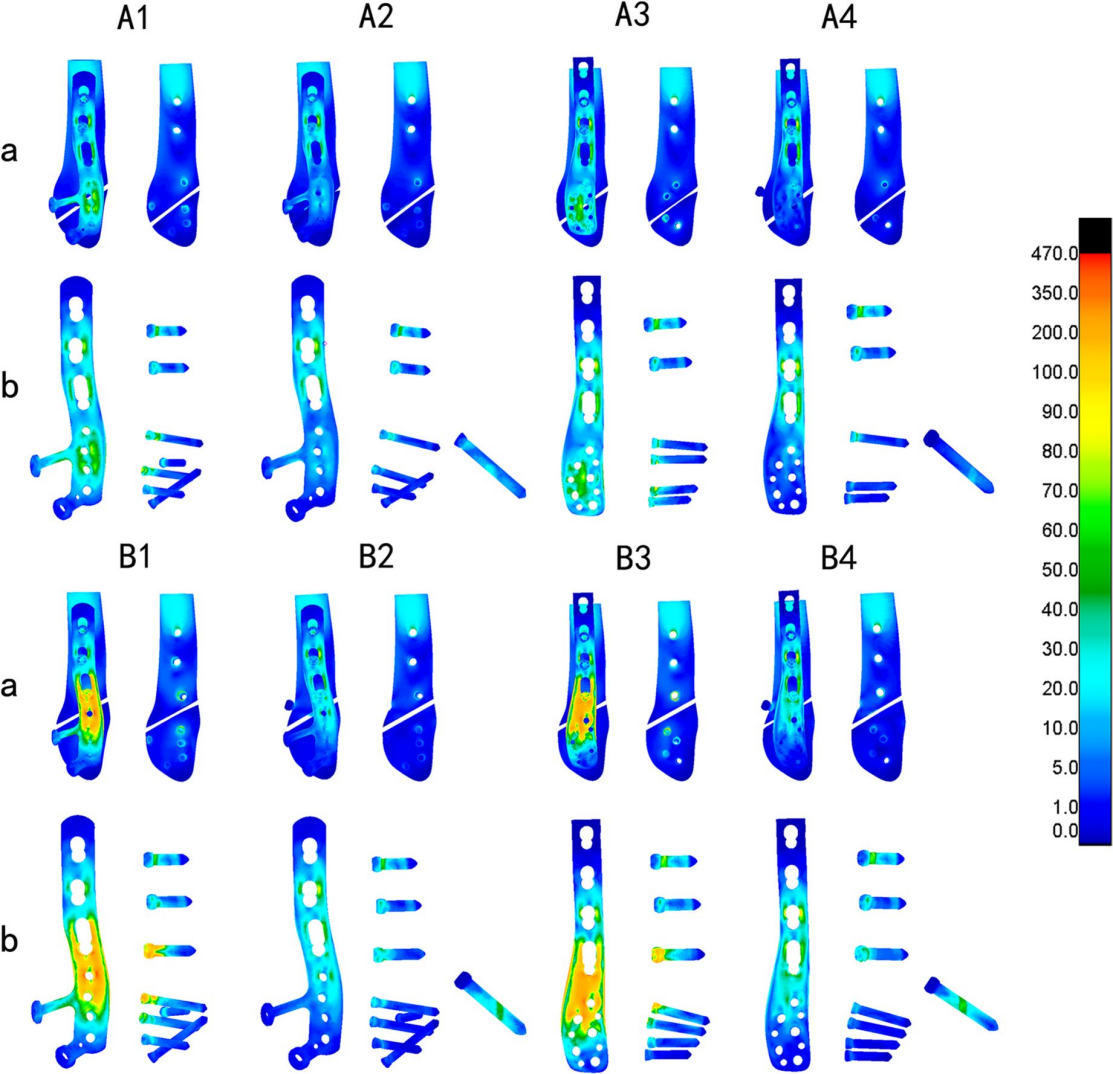
Von Mises Stress (VMS) distribution varied among different fracture types (Danis-Weber A and B) and fixation/implant methods (FMLP, CLP with or without an interfragmentary screw) in the position of one foot. A1: FMLP fo the type A fracture model. A2: FMLP plus an interfragmentary screw for the type A fracture model. A3: CLP for the type A fracture model. A4: CLP plus an interfragmentary screw for the type A fracture model. B1: FMLP only for the type B fracture. B2: FMLP plus an interfragmentary screw for the type B fracture. B3: CLP only for the type B fracture. B4: CLP plus an interfragmentary screw for the type B fracture. a. VMS distribution cloud diagram of the overall structure and fibula. b. VMS distribution cloud diagram of plate, screws, and interfragmentary screw

**Fig. 5:**
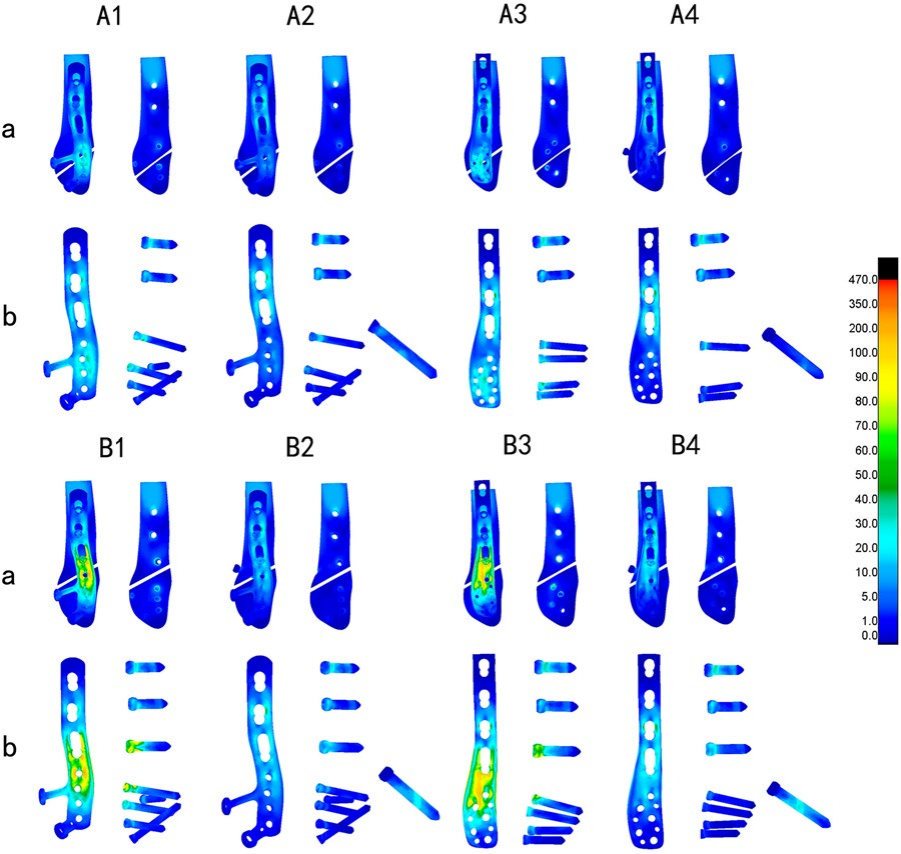
Von Mises Stress (VMS) distribution varies concerning different fracture types (Danis-Weber A and B) and fixation/implant methods (FMLP, CLP with or without interfragmentary screw) in the two-foot standing position. A1: FMLP for the type A fracture model. A2: FMLP plus an interfragmentary screw for the type A fracture model. A3: CLP for the type A fracture model. A4: CLP plus an interfragmentary screw for the type A fracture model. B1: FMLP only for the type B fracture. B2: FMLP plus an interfragmentary screw for the type B fracture. B3: CLP only for the type B fracture. B4: CLP plus an interfragmentary screw for the type B fracture. a. VMS distribution cloud diagram of the overall structure and fibula. b. VMS distribution cloud diagram of plate, screws, and interfragmentary screw

**Fig. 6:**
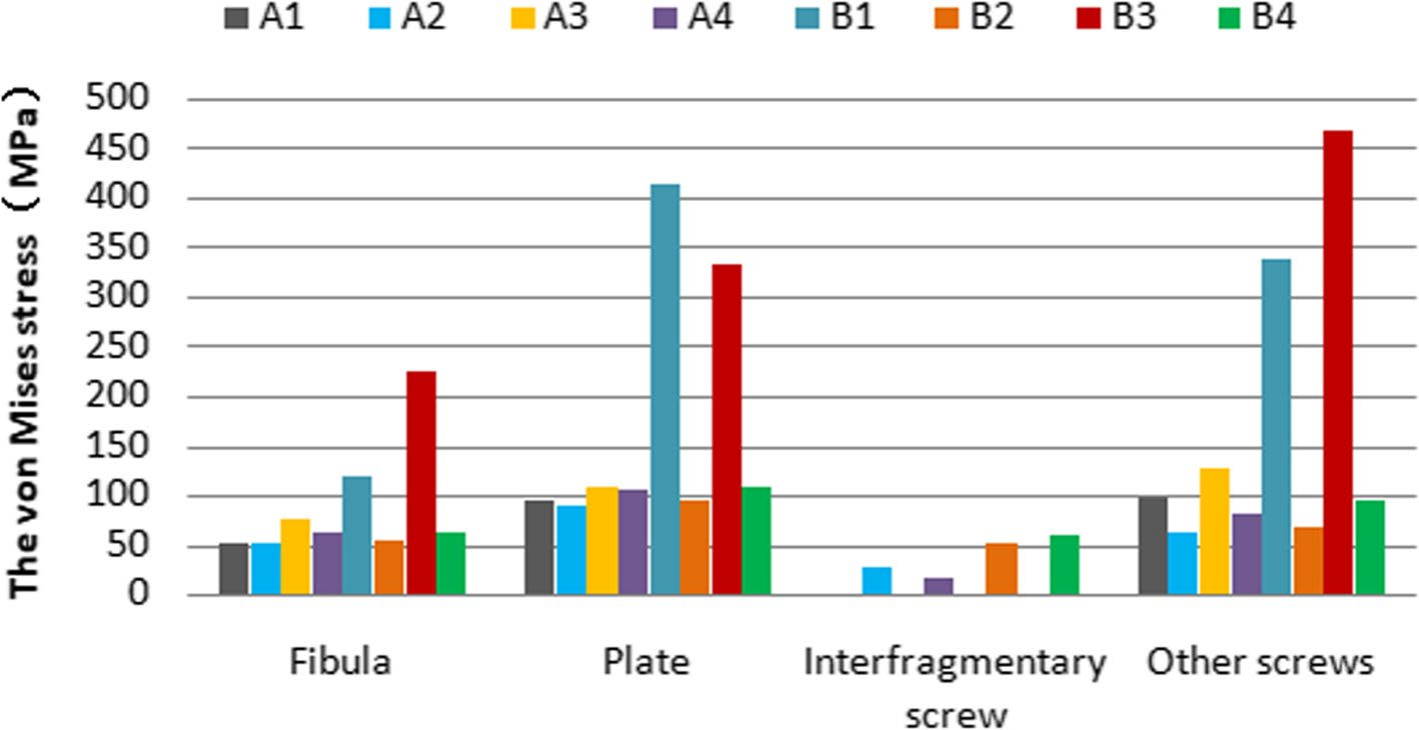
PeakVon Mises Stress (VMS) varies among different model groups representing Fibular Medial Locking Plate (FMLP), Conventional Locking Plate (CLP), with or without an interfragmentary screw for the fixation of Danis-Weber A and B fractures in the one-footed standing position

**Fig. 7:**
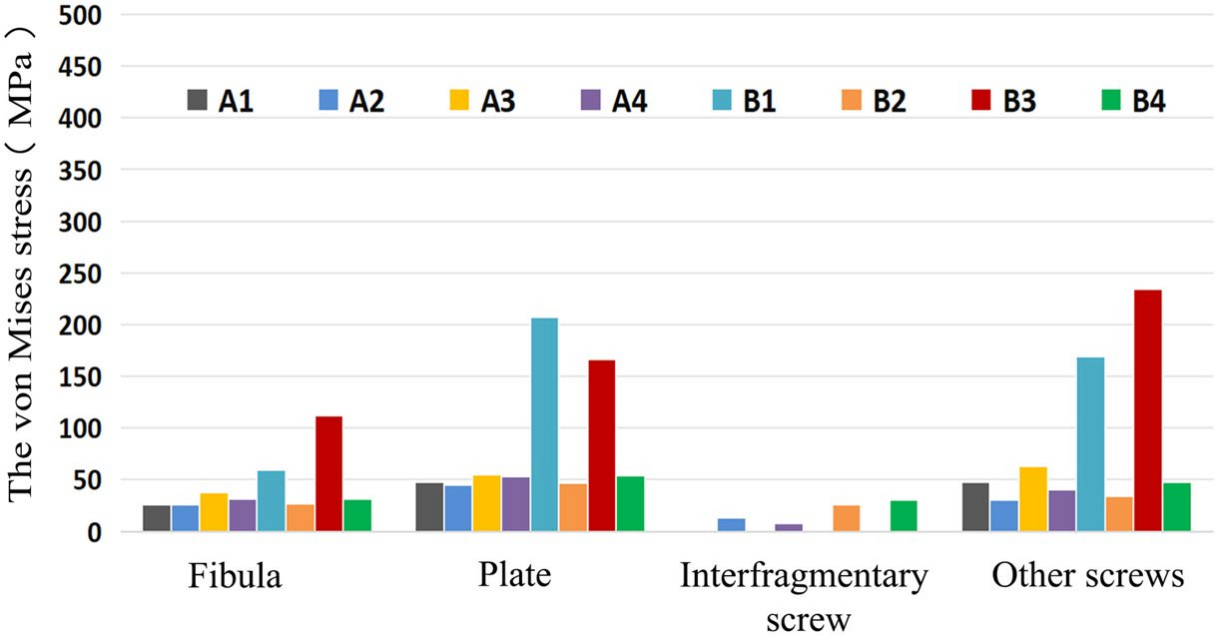
Peak Von Mises Stress (VMS) varies among different model groups representing FMLP, CLP, with or without an interfragmentary screw for the fixation of Danis-Weber A and B fractures during the two-leg standing position

The nephograms illustrating the von Mises stress (VMS) distribution for distinct fracture types (Danis-Weber A and B) and fixation/implant methods (FMLP, CLP with or without interfragmentary screw) under the loading conditions of 140 N and 70 N are depicted in Figs. 4 and 5, respectively. The peaks of VMS for the fibula, the plate, interfragmentary screw, and other screws are documented in Table 3 and Figs. 6, 7.

**Table 3:** Peak VMS distribution (MPa) among different implants and fracture model groups in distal fibula

Models	A1		A2		A3		A4		B1		B2		B3		B4	
Loading(N)	**140**	**70**	**140**	**70**	**140**	**70**	**140**	**70**	**140**	**70**	**140**	**70**	**140**	**70**	**140**	**70**
Fibula	51.9	25.9	51.8	25.9	75.4	37.7	62.5	31.2	118.1	59.0	54.2	27.1	223.9	111.9	62.3	31.2
Plate	94.6	47.3	89.0	44.5	109.4	54.7	106.0	52.9	414.1	206.9	93.4	46.7	333.0	166.4	107.9	53.9
Interfragmentary screw	/	/	26.4	13.2	/	/	15.1	7.5	/	/	51.4	25.7	/	/	60.7	30.4
Other screws	96.5	47.8	61.3	30.6	126.7	63.3	80.1	40.0	338.9	169.4	68.6	34.3	468.9	234.3	95.5	47.7

**Fig. 8:**
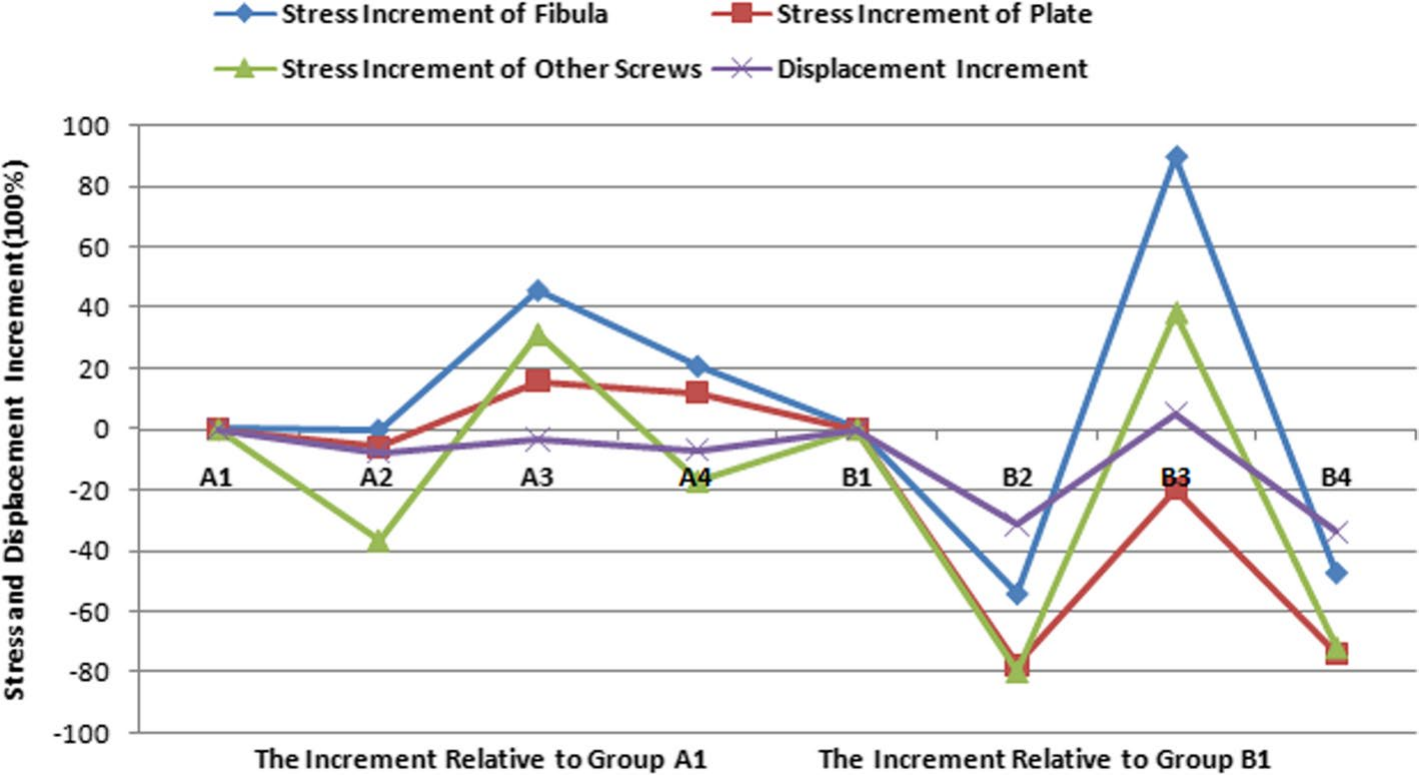
Stress peaks and the displacement relative increment (%) of the distal fibula fractures are observed with different internal fixation groups during one-footed standing position

Additionally, the nephrograms capturing VMS distribution for different fracture types (Danis-Weber A and B) and fixation/implant methods (FMLP, CLP with or without interfragmentary screw) under consistent loading conditions were recorded. In the Danis-Weber A fracture model group, the FMLP with an interfragmentary screw fixation exhibited the lowest peak VMS values: 51.9 MPa in the fibula, 89.0 MPa in the plate, and 61.3 MPa in the screws for simulating single-leg conditions. Under two-leg standing conditions, these peak VMS values decreased to 25.9 MPa in the fibula, 44.5 MPa in the plate, and 30.6 MPa in the screws, respectively. This pattern was consistently observed, as the peak VMS values of the fibula, plate, and screws were consistently lower in the Danis-Weber A fracture groups compared to the type B fracture groups with the same implants. Irrespective of the interfragmentary screw usage, under identical fracture type conditions, the stress peak values of the fibula were lower in the FMLP fixation groups than in the CLP fixation groups. The introduction of an interfragmentary screw at the oblique fracture site resulted in a significant reduction in peak VMS values for the fibula, plate, and screws, particularly for type B fractures. This observation suggests that the FMLP and CLP fixation models with an interfragmentary screw effectively disperse the mechanical loads. The incremental change in stress peak for different fixation model groups relative to groups A1 and B1, respectively, is depicted in Fig. 8.

### The displacement changes

**Fig. 9:**
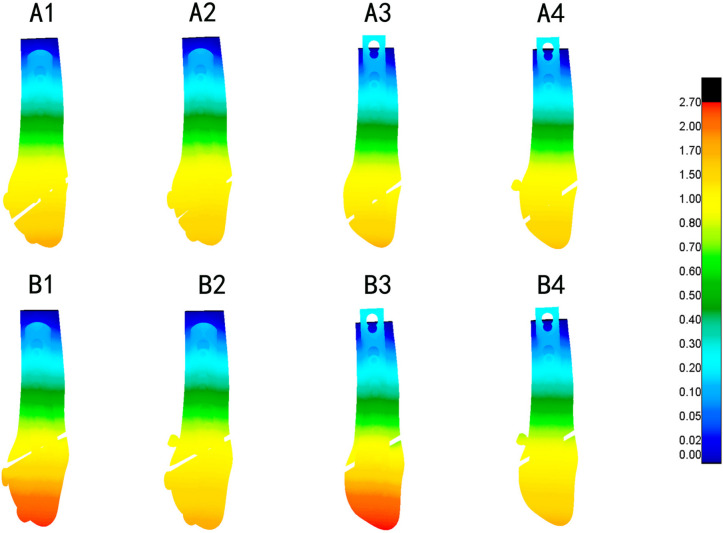
Changes in the overall structural displacemenare evident in various model groups, including FMLP, CLP, with or without an interfragmentary screw, for the fixation of Danis-Weber A and B fractures during one-leg standing position

**Fig. 10:**
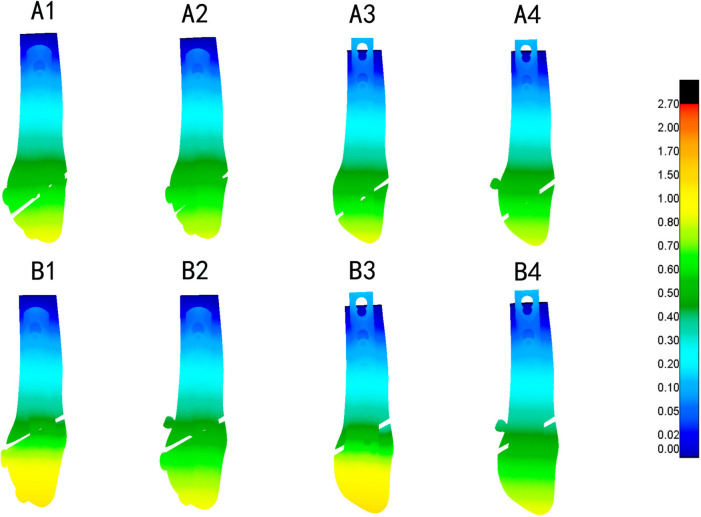
Changes in fracture displacement are observed among different model groups, including FMLP, CLP, with or without an interfragmentary screw, for the fixation of Danis-Weber A and B fractures during two-leg standing position

During single-leg stance, the overall structural maximum displacements of the fragment-implant assembly displacement for Weber-A fractures on the lateral malleolus, using different fixations/implants (FMLP, CLP with or without an interfragmentary screw), ranged from 1.61 to 1.74 mm, while for Weber-B fractures, it ranged from 1.69 to 2.54 mm (Table 4, Fig. 9). Similarly, during bipedal stance, the overall structural maximum displacements of the fragment-implant assembly displacement for Weber-A fractures on the lateral malleolus, employing different fixations/implants (FMLP, CLP with or without an interfragmentary screw), ranged from 1.61 to 1.74 mm, while for Weber-B fractures, it ranged from 1.69 to 2.54 mm (Table 4, Fig. 10). For a direct comparison of the overall structural maximum displacements changes among the eight groups with various internal fixation and fracture models, the displacement increment percentage for each overall structure was calculated relative to A1 and B1 under the same fracture type (Table 4, Fig. 8). In the context of the same type of distal fibula fracture, the overall structural maximum displacement increment in the Weber-B group compared to group B1 was more pronounced than that in the Weber-A group compared to group A1. Specifically, the Weber-B group exhibited a maximum displacement increment of -33.5% compared to group B1 for different fixation/implant methods, while the maximal displacement increment in the Weber-A group was -7.5% compared to group A1. These results indicate that interfragmentary screw fixation contributes to a reduction in the displacement of oblique fractures (Table 4, Fig. 8).

**Table 4:** Displacement (mm) and relative increment (%) in the distal fibula fractures with different internal fixation groups in one-footed and two-footed standing

Models	A1		A2		A3		A4		B1		B2		B3		B4	
Loading(N)	**140**	**70**	**140**	**70**	**140**	**70**	**140**	**70**	**140**	**70**	**140**	**70**	**140**	**70**	**140**	**70**
Displacement (mm)	1.74	0.87	1.61	0.80	1.68	0.84	1.62	0.81	2.54	1.27	1.75	0.87	2.67	1.34	1.69	0.85
Relative increment (%)	0	0	-7.5	-8.0	-3.4	-3.4	-6.9	-6.9	0	0	-31.1	-31.5	5.1	5.5	-33.5	-33.1

In all eight models, the maximum displacements of the fibula fracture fragment, the internal fixation, and the overall structural fragment-implant assembly were consistently observed at the distal end of the lateral malleolus. Furthermore, the displacement demonstrated a gradual decrease from distal to proximal locations, as depicted in Figs. 8, 9, 10. Additionally, the peak micromotion observed in both the fibula fracture fragment and the overall structural fragment-implant assembly consistently remained lower when an interfragmentary screw fixation was employed compared to the group without an interfragmentary screw. This difference was particularly pronounced in type B fractures.

## Discussion

The distal fibula plays a crucial role in ankle stability, and fractures in this region pose challenges for plate osteosynthesis due to the anatomical morphology characterized by a triangular or pyramidal shape and the limited availability of screws for fixation in the short distal end segment. Additionally, the surgical approach is further complicated by limited soft tissue coverage in this region. The critical positioning of the plate on the lateral malleolus can be categorised into two primary methods: lateral and posterior application. While the posterior plating fixation technique offers some anti-sliding stability for the distal fibula, it may cause discomfort or irritation to peroneal tendon lesions [37]. In a lateral plate construct, it becomes essential to employ unicortical screws to prevent penetration into the articular surface of the lateral malleolus. Traditional constructs, however, permit only two screws to be placed into the distal fragment, potentially lacking sufficient stability, particularly in the cases of Danis-Weber A fractures with a very short distal fragment. Locking plate fixation proves beneficial for providing angular stability in treating metaphyseal fractures, such as distal fibular fractures, especially in scenarios of poor bone quality or a small distal fragment. Kim et al. conducted a biomechanical cadaveric study revealing that a locking plate construct with two distal unicortical screws exhibited similar mechanical strength to standard plating with three distal screws in mechanical strength [38]. Switaj et al. and Zahn et al. demonstrated that modern lateral locking anatomically contoured plates, featuring an increased number of distal locking screws, imparted significant biomechanical strength compared to nonlocking contoured plates and posterior anti-glide plates. Furthermore, this biomechanical advantage of lateral locking plate fixation was independent of bone mineral density (BMD) [39, 40]. The distal expansion of the lateral locking plate's shape facilitated a large contact area between the plate and bone, enabling the insertion of four or more unicortical locking screws into the distal fragment of the lateral malleolus in Danis-Weber B type fractures, thereby enhancing the construct's biomechanical strength [41]. However, the plate’s large size and bulky profile of the plate may elevate the risk of incision complications related to soft-tissue closure over a bulky lateral locking plate. Orthopedic surgeons must judiciously select a fixation construct that offers optimal stability for the anatomical reduction of the distal fibula while minimising irritation to the lateral skin. The present study demonstrates that the novel FMLP locking plate, positioned on the posterior lateral ridge of the distal fibula, allows for a greater number of screws through low-profile two wings for fixation in distal fractures. Thus, it is speculated that the FMLP avoids irritation of the peroneal tendon and provides a similar posterolateral buttress effect. Furthermore, in comparison to the lateral locking plate, the FMLP has a lower subcutaneous hardware profile and a reduced lateral plate-bone contact area.

With advancing technology, Finite Element Analysis (FEA) has the capability to simulate and perform comparative biomechanical analyses for complex fractures among different fixation methods, eliminating the limitation of the lack of cases [42]. The adopted displacement analysis technique and von Mises stress (VMS) distribution technique in FEA enable the quantification of independent displacement and peak VMS values for each fragment and implant. Independent motion and stress peaks in bone fragments and implants are highly undesirable in fracture consolidation.

The results of our qualitative and quantitative simulations showed that the VMSs on the screws and fibula during standing on one foot ranged from 61.3–468.9 MPa and from 51.8–223.9 MPa, respectively. These findings align with those of Marvan et al. [8]. Within the range of one order of magnitude variation, the boundary conditions of our biomechanical finite element simulation of slow walking were in line with the actual loading mechanisms. The stress peaks of the bone, plates, and screws calculated for the eight groups of fibula internal fixation in our study were within the yield strength range of their respective materials, and no screws or plates were expected to be damaged at the time of testing. The yield strength of the titanium alloy (Ti-6A1-7NB) material used was approximately 817 MPa [43], and this level of strength could be achieved by reasonably increasing the number of screws used.

As indicated in Tables 3, 4 and Figs. 6, 7, 8, the FMLP models offer greater stability than the CLP models, with the enhanced stability attributed to additional interfragmentary screws. Although the number of screws used for distal fragment fixation in type A fractures was lower than that for type B fractures in our study, we observed that the stress on the fibula, plate, and screws was lower in type A fractures than in type B fractures with the same locking plate fixation. The results of our parametric FE analysis may be influenced by the loading on the talofibular articular contact surfaces at the distal end of the fibula. Specifically, the type A fracture models were situated in realistic loading contact areas, as depicted in Fig. 3. This allowed for partial loading force to be transferred through the proximal part of the fracture, explaining the differences in stress between type A and type B fractures with the same locking plate fixation. As described previously, Weber A fractures of the lateral malleolus are more stable than type B fractures [44, 45]. In the current study, we found evidence suggesting that FMLP provides better biomechanical stability than CLP for the fixation of Weber A fractures, even without interfragmentary screws. Additionally, FMLP was associated with a reduced risk of implant failure.

The technique of employing an interfragmentary lag screw technique has been utilised to augment compression and stability in instances of uncomplicated oblique fibular fractures. As a consequence, the conventional clinical approach for lateral malleolar fractures entails utilising either a neutralisation plate or a locking plate in combination with lag screw fixation. Mechanical experiments conducted previously have illustrated that the technique of employing a locking plate in combination with lag screw fixation imparts superior stability in comparison to the application of a lateral neutralisation plate in tandem with a lag screw, as well as antiglide plating construct involving a lag screw for Weber-B distal fibular fractures [16, 39, 40]. Recent biomechanical investigations have suggested that the intrinsic stiffness of the locking plate construct is minimally affected by the addition of a lag screw. However, the purchase of an interfragmentary screw may introduce data dispersion, thereby complicating the evaluation of the mechanical properties of various plate [15, 40]. Our finite element mechanics study aimed to analyse the effects of different locking plates, with or without interfragmentary screws, on Weber-A and B lateral malleolar fractures. As illustrated in Table 3 and Figs. 4, 5, 6, 7, 8, stress was dispersed by the interfragmentary screw, resulting in a noteworthy decrease in stress on the fibula, plate, and screws in models with an interfragmentary screw, particularly for type B fractures. The outcomes of the present study demonstrated that the addition of an interfragmentary screw can enhance the strength of the locking plate construct and reduce the peak value of VMS on the fibula in a simple lateral malleolus oblique fracture. As depicted in Table 4 and Fig. 8, the incorporation of an interfragmentary screw led to a reduction in the displacement of the oblique fracture, thereby expediting the fracture healing by imparting stability. Thus, we posit that interfragmentary screw fixation, regardless of employing LCP or FMLP constructs, can confer fixation stability in simple oblique fractures of the lateral malleolus. Furthermore, the combination of FMLP and an interfragmentary screw fixation can establish a robust and stable mechanism.

Nevertheless, the current study possesses has several limitations that necessitate consideration to avoid overstating the findings. Firstly, owing to the intricacies associated with establishing and assessing fracture models of the lateral malleolus, the involvement of ankle ligaments and muscle structure was not taken into account in this investigation. Although this simplified loading simulation model proves valuable, it may not offer the most advanced loading analysis. Secondly, Weber A fractures are conventionally regarded as length-stable transverse fibular fractures. However, in our type A fracture model, we intentionally introduced an oblique fracture to augment fracture instability and assess the impact of an interfragmentary screw on fracture alignment. Thirdly, limiting the analysis to a young male volunteer might be considered a constraint in this study, given that this fracture type also significantly affects postmenopausal women due to its bimodal nature. While our objective was to compare different models under the uniform bone quality conditions, and our results remain credible and objective within this framework, it is essential to note that osteoporosis significantly influence the biomechanics of internal fixation. Therefore, further investigations exploring these dynamics in populations with diverse bone qualities, including postmenopausal women, are warranted. Lastly, since the values assigned to bone and material properties were derived from existing literature rather than individual measurements, and the parameters of implanted bone and material may undergo changes post internal fixation, this study should be regarded as a preliminary exploration into the analysis of internal fixation in distal fibular fractures. This experiment is exclusively conducted using the finite element method, and additional experimental research on cadaver biomechanics is imperative for validation. Nonetheless, to acquire evidence-based insights into the failure rate of internal fixation, complication rate, and ankle function score, supplementary clinical observational studies are imperative to assess the efficacy and safety of FMLP.

## Conclusions

In conclusion, present finite element analysis (FEA) demonstrates that, in comparison to conventional locking plates (CLP), the novel fibular medial locking plate (FMLP) manifests improved mechanical characteristics in Danis-Weber A and B lateral malleolus fractures. Furthermore, the amalgamation of an interfragmentary screw with a locking plate construct imparts enhanced stability for a simple oblique distal fibular fracture. Consequently, the FMLP emerges as a potential alternative for addressing lateral malleolus fractures from a biomechanical standpoint. Nevertheless, these findings require validation through additional clinical studies.

## Data Availability

All data generated or analysed during this study are included in this published article.

## Abbreviations

FMLP: Flank multiaxial locking anatomic plate
CLP: Conventional distal fibula locking plate
FEA: Finite element analysis
AO/OTA: Arbeitsgemeinschaft für osteosynthesefragen foundation/orthopaedic trauma association
ORIF: Open reduction and internal fixation
CT: Computed tomography
DICOM: Digital imaging and communications in medicine
VMS: Von Mises stress
BMD: Bone mineral density
